# Description of a Hybrid Mindfulness-Integrated Multidisciplinary Workplace Weight Management Intervention Module ‘Mind-SLIMSHAPE’ Using the TIDieR Checklist

**DOI:** 10.3390/nu14153140

**Published:** 2022-07-29

**Authors:** Siti Munirah Abdul Basir, Zahara Abdul Manaf, Fatin Hanani Mazri, Arimi Fitri Mat Ludin, Suzana Shahar, Mohd Rizal Abdul Manaf

**Affiliations:** 1 Centre for Healthy Aging and Wellness and Dietetic Program, Faculty of Health Sciences, Universiti Kebangsaan Malaysia, Jalan Raja Muda Abdul Aziz, Kuala Lumpur 50300, Malaysia; sitimunirah.abdulbasir@gmail.com (S.M.A.B.); fatinhananimazri@gmail.com (F.H.M.); suzana.shahar@ukm.edu.my (S.S.); 2 Centre for Healthy Aging and Wellness and Biomedical Science Program, Faculty of Health Sciences, Universiti Kebangsaan Malaysia, Jalan Raja Muda Abdul Aziz, Kuala Lumpur 50300, Malaysia; arimifitri@ukm.edu.my; 3 Department of Community Health, Faculty of Medicine, Universiti Kebangsaan Malaysia, Jalan Yaacob Latif, Bandar Tun Razak, Cheras, Kuala Lumpur 56000, Malaysia; mrizal@ppukm.ukm.edu.my

**Keywords:** workplace intervention, obesity, mindful eating training, description, design, TIDieR

## Abstract

Published reports of workplace-based weight management interventions are often poorly described and are focused on dietary, physical, and behavioral management. These strategies are often unsustainable and only have short-term effectiveness. The Mind-SLIMSHAPE^TM^ is a mindfulness-integrated multidisciplinary intervention developed to address overweight and obesity problems among desk-bound employees while improving weight-related behavior through mindfulness meditation and mindful eating exercises. The integration of mindfulness and mindful eating aims to improve the individual’s focus on the present and heighten their sensitivity towards internal and external eating cues. The aim of this article is to describe the Mind-SLIMSHAPE^TM^ intervention program using The Template for Intervention Description and Replication (TIDieR) checklist. The Mind-SLIMSHAPE^TM^ module is a 24-week intervention program that was delivered in a quasi-experimental study among employees with BMI ≥ 25.0 kg/m² in a selected higher learning institution. The module was delivered via hybrid sessions that included both face-to-face and virtual online sessions. The novelty of our description includes summaries of each intervention component with its intensity, details of the theory grounded for this program, and the rationale for the intervention components. The Mind-SLIMSHAPE^TM^ module is ready to be implemented and replicated in a similar setting with possible refinement and enhancement of the mindfulness and mindful eating elements.

## 1. Introduction

Obesity is a worldwide public health concern that requires effective intervention programs that target modifiable health-related behaviors aligned with relevant groups, socio-demographic, and psychosocial characteristics. The global prevalence of overweight and obesity nearly tripled between 1976 and 2016, with 52% comprising adults aged 18 years and above and more common among women [1]. In Malaysia, the prevalence of overweight and obesity in the government and private work sectors showed an increasing trend with a 5% increment from 2011 to 2019 [2,3]. Obesity has been linked to increased risk of chronic diseases (e.g., type 2 diabetes, cardiovascular heart diseases, cancers) [4,5,6], musculoskeletal disorders [7], and emotional health decline (e.g., depression, anxiety) [8,9]. It imposes a financial burden on employees and employers as it increases absenteeism [10], healthcare cost [11], decreases productivity [12], and negatively impacts quality of life [13]. This necessitates the need for the development, implementation, and evaluation of effective workplace weight management strategies.

Common weight management strategies include calorie restriction, engagement in physical exercise, behavioral therapy, and drug therapy [14]. However, these strategies are often reported to be unsustainable in the long term. A multicenter randomized controlled study in the United States reported that participants regained at least half of their initial weight loss after five years [15]. A randomized crossover trial in Japan found no significant difference in mean body weight after one year of a worksite-based weight loss program [16]. Mindfulness-based interventions have been promoted as a behavioral change approach for a more sustainable weight loss and minimizing overweight and obesity [17]. It has been theorized that mindfulness promotes adaptive self-regulation, which is the key to maintaining long-term eating habits [18]. The concept of mindfulness originates from Buddhism [19]. It refers to the ability to be open, accepting, and present in the moment [20]. Mindfulness training is commonly defined as an intervention that aims to foster non-judgmental and moment-to-moment awareness of the present experience. The use of mindfulness-based cognitive behavioral interventions such as Mindfulness-Based Stress Reduction (MBSR), Mindfulness-Based Cognitive Therapy (MBCT), and Mindfulness-Based Eating Awareness Training (MB-EAT) has been recommended to manage the physical and psychological health of individuals with obesity in a clinical context [21].

The self-regulation theory proposes that the regulation of internal physiologic processes depends on the ability to observe internal responses [22]. In eating, it refers to the ability to respond to internal cues (e.g., hunger and satiety) without being overly influenced by external cues (e.g., food cues or emotional states) [23]. Obesity-related eating behaviors can be partially explained by the inability to identify and respond to internal cues of hunger and satiety [23,24]. It is correlated with increased episodes of overeating [25] and a greater risk for weight gain [26]. The practice of mindfulness can be applied to the reduction of food cravings, portion control, and body weight [27]. Mindful eating is about being non-judgmental and aware of (one’s) physical and emotional sensations while eating or in a food-related environment [20]. It increases one’s sensitivity to the physical signs of hunger, satiety cues, the pace of eating, food environment, and food characteristics [27]. These cues are fundamental to self-regulation and control the urge to consume high-calorie foods. Individuals practicing mindful eating have lower problematic eating behaviors, decreased sugar consumption, and consume smaller serving sizes of energy-dense foods [28,29,30]. In addition, mindfulness has been shown to improve psychological outcomes, such as anxiety and stress, and improve weight-related negative eating behaviors, such as stress and emotional eating.

Setting-based approaches are proposed as a cost-effective policy for reducing obesity prevalence. In particular, workplace interventions addressing diet, physical activity, and the work environment have been emphasized by WHO [31]. Mindful eating intervention studies have mostly been conducted among general populations in developed countries, focusing on dietary-related outcomes [32], however the impact on cardiometabolic risk factors has been inconclusive [33]. In Malaysia, workplace obesity intervention studies are still limited. Previous workplace-based interventions focused on either multidomain interventions that included dietary, physical and behavioral management [34,35], physical activity [36], or lunch meal replacement [37]. In another study among university employee, mindful eating was included as part of an intervention. However, the details of its mindful eating component were not clearly outlined [38]. The delivery of the aforementioned interventions was either face-to-face or online via the web. To this date, remote interventions delivered through online platforms such as Zoom and Facebook Live have yet to be reported. In view of the high prevalence of obesity among employees and limited reported local workplace intervention, the need for specialized intervention programs is paramount. Thus, a mindfulness-based behavioral and multidisciplinary structured workplace weight-loss intervention (Mind-SLIMSHAPE^TM^) is being proposed to combat obesity at the workplace among multiethnic Malaysian adults. The purpose of this article is to describe the development and intervention design of Mind-SLIMSHAPE^TM^ using a 12-item Template for Intervention Description and Replication (TIDieR) checklist (Hoffmann et al., 2014). TIDieR is an extension of the Consolidated Standards of Reporting Trials (CONSORT) [39] and Standard Protocol Items (SPIRIT) [40] statements, which were developed to guide and improve the quality of reporting on interventions by ensuring transparency [41]. The aim of the intervention study is to investigate its effectiveness among employees with overweight and obesity.

## 2. Description of Mind-SLIMSHAPE^TM^ Using the TIDieR Checklist

The study procedures described below were approved by the Secretariat of Medical Research and Innovation, Faculty of Medicine, Universiti Kebangsaan Malaysia, dated 1 July 2019 (UKM FPR.4/244/DCP-2018-005/1). Written consent was obtained from each participant before the intervention.

*Item* *1* *BRIEF NAME: Provide the name or a Phrase That Describes the Intervention*

Mind-SLIMSHAPE^TM^ is an acronym for the Mindful Eating and SLIM with Structure Healthy Activity, Psychology, and Eating Program. It depicts a multidomain intervention with the integration of mindfulness and mindful eating practices among employees with overweight and obesity.

*Item* *2* *WHY: Describe any Rationale, Theory, or Goal of the Elements Essential to the Intervention*

The original SLIMSHAPE^TM^ intervention program was created based on the six primary constructs of the Health Belief Model (HBM) [42]: perceived severity (an individual’s perception of the seriousness and potential consequences of the condition), perceived susceptibility (an individual’s assessment of their risk of getting a disease or condition), perceived benefit (an individual’s belief about whether the recommended behavior will reduce the risk or severity of impact), perceived barrier (an individual’s assessment of the difficulties and cost of adopting behaviors), cue to action (the internal or external motivations promoting the desired behavior), and self-efficacy (an individual’s belief about their capabilities to successfully perform a new health behavior) [34,35]. This model explains one’s action to prevent, screen for, or control illness conditions. For example, individual consultation based on anthropometry and biochemical assessment provide a view of the seriousness of one’s condition with regard to having a higher BMI, thus setting cues for action. In addition, continuous weight monitoring aims to enhance an individual’s self-efficacy and perceived benefit of weight-related behavioral changes.

The Mind-SLIMSHAPE^TM^ was developed to address the common challenges in weight management: the sustainability of lost weight and a healthy lifestyle beyond the intervention period. Obesity interventions often result in progressive regain after a period of rapid weight loss and weight plateau [43]. Mindfulness and mindful eating training are integrated as an adjunct strategy to the existing SLIMSHAPE^TM^ program to further support and enhance weight-loss success. The goal of mindful eating is to address the chronic imbalance of oversensitivity to non-nutritive food cues and the lack of utilization of physiologically based hunger and satiety cues [44]. This internal/external response imbalance is hypothesized to be heightened in the obese population. Furthermore, frequent dieting further disengages this population from the appropriate response to the internal cues of hunger and satiety, making them more susceptible to triggers of overeating. Incorporating other social cognitive constructs such as social support and goal setting [45], Mind-SLIMSHAPE^TM^ aims to blend educational and experiential learning by implementing a structured mindfulness-integrated intervention to address overweight and obesity problems among desk-bound employees. Anticipated outcomes and impacts of the intervention include changes in body composition and overall improvement in short-term cardiometabolic health, as well as long-term sustainability of healthy behavior and mindfulness.

*Item* *3* *and* *4* *WHAT: Describe Materials and Procedures*

The Mind-SLIMSHAPE^TM^ is a 24-week weight-loss intervention program that integrates the concept of mindfulness and mindful eating by adopting and adapting the mindfulness-based eating awareness training (MB-EAT) module [44,46] into an existing weight-loss intervention module (SLIMSHAPE^TM^) developed by Rusali et al. [34,35]. Table 1 illustrates the comparison between the SLIMSHAPE^TM^ and Mind-SLIMSHAPE^TM^ programs. SLIMSHAPE^TM^ and Mind-SLIMSHAPE emphasize dietary and physical activity modifications as the core intervention. The Mind-SLIMSHAPE^TM^ program includes mindfulness and mindful eating components as additional behavioral modifications aimed to enhance weight-loss success and ensure long-term sustainability.

**Figure 1:**
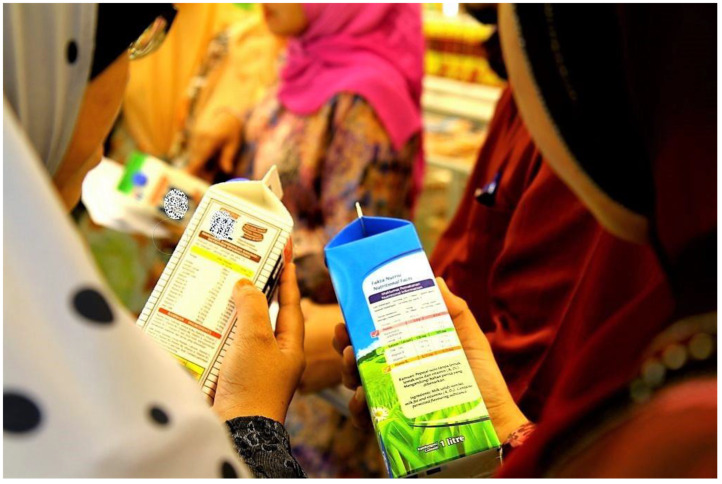
Understanding nutrition label reading during ‘Supermarket Tour and Sweep’ session.

Table 2 summarizes the Mind-SLIMSHAPE^TM^ intervention program. The intervention components include dietary management, physical activity and exercise, mindfulness, and social cognitive therapy. These components are delivered through didactic talks, interactive activities, and group exercises. A total of 10 sessions are allocated to deliver nutrition-related weight-loss topics such as dietary modifications, physical activity and exercise, the importance of sleep, and healthy packed meals. These talks last approximately 60 to 90 min per session. Group exercises vary from low impact (relaxing yoga and resistance training) to high impact exercises (HIIT and circuit training) and are conducted for at least 45 min at the end of the session. Interactive activities that last approximately 30 to 120 min provide hands-on applications of related topics to enhance the participants’ understanding. For example, the ‘Supermarket Tour and Sweep’ session provides experiential learning related to calories and nutrients in various foods and beverages (Figure 1). This activity is aimed at improving participants’ grocery-shopping skills to purchase better and healthier choices.

Two forms of meditations are used in the Mind-SLIMSHAPE^TM^ program: general mindfulness (breath awareness) meditation and guided eating meditations. Breathing awareness meditation aims to train one’s attention and awareness to the present—breathing patterns, thoughts, emotions, and bodily sensations—with non-judgmental observation and less reactivity. Eating meditations help to elevate individual awareness of the experiences of hunger, fullness, taste, and food choice through mindfully eating small amounts of challenging foods such as crackers, chocolates, and cakes [23]. In Mind-SLIMSHAPE^TM^, breathing meditation is conducted at the beginning of each mindfulness session while eating exercises are conducted at the end, followed by ten minutes of reflection. In addition, home meditation practices were encouraged to improve mindfulness and mindful eating skills.

The outcome measures were assessed at pre-, post-, and eight-month follow-up intervals to assess the effectiveness and sustainability of the Mind-SLIMSHAPE^TM^ program (Table 3). Before the intervention, participants completed the health screening questionnaire which consisted of a social-demographic profile, risk evaluation, and health and nutrition status (smoking, alcohol, diet supplementation, etc.,) to qualify for the program. All questionnaires were self-administered except for the Diet History Questionnaire (DHQ) [47] and the Global Physical Activity Questionnaire (GPAQ) [48].

**Table 2:** Summary of Mind-SLIMSHAPE^TM^ module contents.

Module	Program	Summary of Contents	Intensity (min)	Delivery Mode
**Dietary management**	Calorie deficit [49,50]	Women: 1200–1500 kcal/day Men: 1600–1800 kcal/day	30	FTF
	**Lecture**			
	Obesity and its implications	An introduction to obesity and its implications for health and quality of life	120	FTF
	Understanding body composition and energy balance	Introduction to body composition and energy balance	90	FTF
	Food exchange: How much I can eat?	Application of food exchange in daily meal planning to meet the energy requirement	120	FTF
	Healthy eating guide	A guide to healthy eating practices with menu examples and alternatives	120	FTF
	Fat detective	Understanding dietary fats and application of fat exchanges to estimate total fat content in foods	90	VO
	Slim and fit throughout Ramadan fasting	Tips and guides for dietary and physical exercise management during the fasting month	90	VO
	Sleep and weight	Understanding the role of sleep for successful weight loss	90	VO
	Packed meals for work	Tips and guides to prepare healthy packed meals for work	90	VO
	Weight loss traps	Identifying traps for successful weight loss and ways to overcome them	90	VO
	Overcoming plateaus	Understanding plateaus in weight loss and ways to overcome it	90	VO
	Calorie traps	Identifying hidden sources of extra calorie consumption in daily activities	90	VO
	**Interactive activities**			
	How many calories do I need?	Determination of an individual’s daily calorie needs	30	FTF
	How much sugar is in your drinks?	An estimation of the amount of sugar in selected sweetened beverages	60	FTF
	Healthy MCO meals	A competition on healthy menu preparation during the MCO	60	VO
	Healthy cooking demonstration	A cooking demonstration on healthy and easy meal preparation	90	VO
	Calorie Tracker and Maze	Estimation of calories in five sets of menus	60	FTF
	Do you know these *ulams* (salad)?	Benefits of *ulams* (salad) ‘Guess and name’ game of 10 types of common *ulams* (salad).	60	FTF
	Supermarket Tour and Sweep	Education on how to read food labels and make healthier food choices.	180	FTF
**Physical Activity and Exercise**	Strength/Resistance Cardio Exercise Flexibility Home/Office Workout	Resistance Tube Exercise Fun Aerobic Exercise, Power Walk, and High-Intensity Interval Training (HIIT) Yoga and Stretching Exercises	390	FTF and VO
**Mindfulness**	**Lectures**			
	Mindful eating 101	An introduction to the concepts of mindful eating	60	VO
	Am I truly hungry?	Awareness of true hunger and fullness	60	VO
	Full but unsatisfied	Awareness of taste and body satisfaction	60	VO
	Mindful eating during festivities	Tips and guides to eating mindfully during festivities	60	VO
	What is with calories? The outer wisdom	Awareness of food energy and balance, connecting outer and inner wisdom	60	VO
	Emotional eating: I am in control	Awareness of emotional eating and ways to balance it	60	VO
	Mindfulness in exercise	Application of mindfulness in physical activities and exercises	60	VO
	**Mindfulness meditation**	Practices of breathing meditation	70	VO
	Mindful eating exercise	Food tasting and appreciation practices: raisin exercise cookies/crackers exercise body scan	45	VO
**Social Cognitive constructs**	Social support: WhatsApp and Facebook group	A medium for information relay, support, and reminder	N/A	N/A
	Self-monitoring	Weekly weight goal and monitoring in the printed weight-loss diary	N/A	N/A
	Motivation	Successful weekly weight loss was awarded an encouragement sticker (one sticker/0.5 kg weight loss) Motivational forum by an alumnus of SLIMSHAPE^TM^	N/A 120	N/A VO

N/A—not applicable; FTF—face-to-face; VO—virtually online.

**Table 3:** Pre- and post-intervention assessments.

Measurements	Description/Equipment	Week 0 Pre-Assessment	Week 25 Post-Assessment	Week 32 Post-Assessment
Informed Consent		√		
Demographic Information		√		
Anthropometrics	Height: using SECA Bodymeter 208 (SECA, Germany) Waist circumference: Lufkin Executive^®^ Diameter Pocket Tape, model W606PM (US)	√		
Weight and Body Composition	Weekly self-monitoring using a bathroom weighing scale	√	√	√
Vital Sign	Blood pressure	√	√	√
Blood Profile	Fasting blood glucose (FBG) HbA1c HOMA IS HOMA IR Lipid profile Uric acid	√	√	√
Stage of Change [51]	Five items assessing the readiness to change for weight management	√	√	√
Process of Change [52]	34 items providing information about the processes of change in weight management	√	√	√
Night Eating Questionnaire (NEQ) [53]	17 items assessing the behavioral and psychological symptoms of Night Eating Syndrome	√	√	√
Malay-translated Mindful Eating Questionnaire (MEQ-M) [54]	28 items assessing the degree of mindful eating behavior	√	√	√
Diet History Questionnaire (DHQ) [47]	7-day information on food intake and usual dietary habits.	√	√	√
Global Physical Activity Questionnaire (GPAQ) [48]	16 items providing information on physical activity in three domains (activity at work, travel to and from places, and recreational activities) as well as sedentary behavior.	√	√	√

*Item* *5* *WHO PROVIDED: Describe the Expertise, Background, and Specific Training Given to Each Category of Intervention Provider*

Mind-SLIMSHAPE^TM^ involved a multidisciplinary team including dietitians, sports science experts, physiotherapists, psychologists, and medical officers. Each intervention session was delivered by an expert in the related field or topics. The interactive sessions were led by an instructor with the assistance of a team of facilitators. The facilitators were involved in the preparation of materials (i.e., booklets or forms) and guided a group of eight participants based on the instructor’s direction. Manual guides and briefings were provided to the facilitators prior to group interactive activities. The mindfulness and mindful eating topics were delivered by one of the dietitians who had formal training in those practices.

*Item* *6* *HOW: Describe the Mode of Delivery of the Intervention*

Mind-SLIMSHAPE^TM^ was designed to be delivered in a hybrid mode, combining face-to-face and live virtual group sessions every week. This mode of delivery was implemented due to the restrictions of face-to-face on-site research activities during the period.

*Item* *7* *WHERE: Describe the Type (s) of Location (s) Where the Intervention Occurred, Including Any Necessary Infrastructure or Relevant Features*

This intervention was delivered in private spaces with access to chairs, tables, and audiovisual equipment for face-to-face sessions. As for live virtual sessions, private spaces with audiovisual equipment and internet connection were utilized. A spacious area is also an important feature for the group exercise session.

*Item* *8* *WHEN and HOW MUCH: Describe the Number of Times the Intervention Was Delivered and Over What Period of Time*

The interventions were executed once a week for 24 consecutive weeks. Each session lasted 2 to 3 h. All activities were conducted from 2 to 5 pm on every Wednesday.

*Item* *9* *TAILORING: If the Intervention Was Planned to Be Personalized, Titrated, or Adapted, then Describe What, Why, When, and How*

Individual dietary modification is one of the components of the Mind-SLIMSHAPE^TM^ intervention. As shown in Table 2, calorie restriction was prescribed based on gender. Individuals’ calorie needs were determined during an interactive activity called ‘How Many Calories do I Need?’. In addition to that, the participants were guided in carbohydrate, protein, and fat exchanges to help them in their daily meal planning based on their calorie needs. In terms of exercise, the instructors provided a few alternatives to exercise forms to accommodate certain physical difficulties, especially for beginners. Participants were also encouraged to exercise based on their capabilities. During the mindful eating exercise, the participants were encouraged to bring food that was personally challenging to them.

*Item* *10* *MODIFICATIONS: If the Interventions Were Modified during the Course of the Study, Describe the Changes (What, Why, When and How)*

The Mind-SLIMSHAPE^TM^ intervention program was initially planned as a 12-week program. However, due to the unforeseen circumstances related to COVID-19, the Mind-SLIMSHAPE^TM^ module was restructured into a 24-week intervention program. The first six sessions were conducted face-to-face and physically on site. During the Movement Control Order (MCO), where movements were limited and social group activities were not allowed, the interventions were delivered virtually and live via online platforms (Zoom and Facebook) for 17 weeks. The last session (week 24) was conducted physically on site as the restrictions were eased in July 2020.

*Item* *11* *&* *12* *HOW WELL: Planned and Actual Fidelity to the Intervention*

Mind-SLIMSHAPE^TM^ is a structured intervention meant for manual execution. It has specific objectives to be achieved in each session delivered via didactic talks and group activities. A pre- and post-session checklist are prepared and completed by the program coordinator to ensure fidelity to the intervention manual.

## 3. Discussion

Research suggests that mindfulness and mindful eating-based intervention could be a practical approach to weight control among adults with overweight and obesity [32]. The non-calorie restriction approach may be a more sustainable adjunct strategy to address long-term weight maintenance after initial weight loss. The Mind-SLIMSHAPE^TM^ is a multidisciplinary approach to integrating mindfulness and mindful eating components for sustainable weight-loss success. To the best of our knowledge, this is the first description of a multidomain program with integration of mindful eating developed to address overweight and obesity among desk-bound employees using the TIDieR checklist. Well-described interventions are important to allow replication and to build on the existing knowledge. Previous reviews found that reporting of interventions in behavioral change research is insufficient [55]. Content descriptions are often brief and vague, using broad categorizations such as ‘behavioral counseling’ or ‘motivational strategies’. Here, we have described the Mind-SLIMSHAPE^TM^ intervention in detail, guided by the TIDieR checklist to address this gap. Our description is novel in that it includes summaries of each intervention content with its intensity, the details of the theory grounded for this program, and the rationale for intervention components. The TIDieR checklist was chosen as a template to describe the intervention as it provides a clear structure and relevant items in describing an intervention.

A successful weight-loss intervention must be engaging and stimulating to enhance learning and understanding. As shown in Table 1, the Mind-SLIMSHAPE^TM^ intervention program was delivered via talks and group activities. The activities were designed to ensure more engagement from the participants. Interactive activities, particularly, allow experiential learning which could help accelerate learning and encourages reflection of the new experience to develop new skills, attitudes, or ways of thinking [56]. In taking one example, participants are allowed to experience the different levels of sweetness based on the number of teaspoons of sugar added to their drink. As sugar intake contributes to their calorie intake, this activity aimed to bring awareness to the level of sweetness and calorie content of sugar-added beverages. In the talks, two-way communication was encouraged through questions and answers, and pop-quiz sessions were conducted to ensure learning objectives were achieved. With respect to the mindful eating component, we would like to suggest that the module be delivered face-to-face and on site to make it more engaging and to enhance participants’ mindful eating experiences. Some of the MB-EAT activity modules such as potluck meals and mindful walking should also be included in the ‘Mindful eating during festivities’ and ‘Mindfulness in exercise’ sessions, respectively. These activities were not included in the present module due to the limitations related to the MCO. The potluck meal eating exercise helps to train individuals to listen and respond to the stomach satiety cues, while mindful walking is an approach to encourage increased physical activity [46].

Our intervention study was confronted with various restrictions due to the unprecedented situations related to the COVID-19 pandemic. A shift of national focus on management and prevention of the COVID-19 outbreak led to a restriction of group activities, face-to-face physical interactions, and movement restrictions. Consequently, any research activities unrelated to COVID-19 were not permitted to be carried out on site. In the wake of the pandemic, ongoing and future clinical research has been revised and restructured to allow for virtual research implementation [57]. In this article, we have proposed and outlined a hybrid mindfulness-based weight-loss intervention module that comprises both virtual and physical sessions to mitigate the impact of the pandemic on the intervention progress. Internet-based intervention is considered to be more effective and cost-saving as it reduces the need to travel for face-to-face workshops and counseling and also reduces the use of printed materials [58,59]. However, this intervention design often has a low engagement and retention rate due to the lack of individual interactions and a limited ability to involve verbal, aural, and physical cues [35,59]. As a solution, a hybrid approach combining both online and FTF interventions might be able to mitigate these limitations while maintaining distinct advantages [59]. Hybrid intervention enables anytime access to the intervention materials and resources which could be of advantage to those participants who are unable to participate during the live or on-site sessions, particularly if it involves working adults. In our case, the recorded sessions were uploaded into our private Facebook group for easier access. Conducting interventions among working adults within their working hours may create an unavoidable conflict of commitment between work and intervention participation. The Mind-SLIMSHAPE program is an example that a workplace-based intervention in the future may adopt a hybrid delivery design to overcome this conflict, if present. Furthermore, the hybrid design may be of more relevant choice as the Work from Home policy is still effective, despite the restriction orders having been lifted.

There are some suggestions to be considered for further improvement in future studies. First, we suggest an assessment of the participants’ digital literacy prior to the interventions. A tutorial guide on the use of these mediums may be provided to those who are less literate. Second, an IT technician is an important resource for online intervention to cater to the internet and IT-related issues, which we lacked during the intervention. Technological/digital literacy is crucial in an online intervention [60]. Lastly, a private space is essential for participants to enable openness in sharing eating experiences during the mindful eating practice without being distracted by the presence of family members or coworkers.

## 4. Conclusions

The increasing prevalence and severity of adult obesity call for an effective and sustainable weight-loss intervention. Because conventional intervention typically results in weight regain, it is important to address other factors such as insensitivity to internal eating cues that could be the root cause of unsustainable weight loss. The description of Mind-SLIMSHAPE^TM^ using the TIDieR framework allows for replication in a similar setting and further refinement of the intervention, particularly on the mindful eating elements. In this article, we provide evidence that weight management intervention programs are still applicable, with the availability of online platforms despite the challenges and pandemic-related restrictions that were faced. Hybrid interventions may still be relevant for a workplace weight management program as the Work from Home policy is still applicable to this date.

## Figures and Tables

**Table 1:** Comparisons between SLIMSHAPE^TM^ and Mind-SLIMSHAPE^TM^ program.

Similarities	
Face-to-face group approach. Involves multidisciplinary interventionists such as experienced dietitians, sports science experts, physiotherapists, and medical officers. Includes dietary and physical activity educational talks and interactive group sessions. Emphasis on a calorie deficit of 500–1000 kcal/day based on the individuals’ daily calorie needs. Delivered weekly.	
Different aspects	
SLIMSHAPE^TM^	Mind-SLIMSHAPE^TM^
Duration: 16 weeks	Duration: 24 weeks
Focuses on dietary calorie modification and exercise training only	Includes mindfulness and mindful eating training
Mode of delivery: Face-to-face	Mode of delivery: hybrid (face-to-face and online)

## Data Availability

Not applicable.
